# Advanced Analysis Techniques for Intra-cardiac Flow Evaluation from 4D Flow MRI

**DOI:** 10.1007/s40134-016-0167-7

**Published:** 2016-05-20

**Authors:** Rob J. van der Geest, Pankaj Garg

**Affiliations:** Division of Image Processing, Department of Radiology, Leiden University Medical Center, Albinusdreef 2, 2333 ZA Leiden, The Netherlands; Division of Biomedical Imaging, Leeds Institute of Cardiovascular and Metabolic Medicine (LICAMM), University of Leeds, Leeds, LS2 9JT UK

**Keywords:** 4D Flow, CMR, Kinetic energy, Vortex, Image processing, Streamlines, Path lines, Flow components

## Abstract

**Purpose of the Review:**

Time-resolved 3D velocity-encoded MR imaging with velocity encoding in three directions (4D Flow) has emerged as a novel MR acquisition technique providing detailed information on flow in the cardiovascular system. In contrast to other clinically available imaging techniques such as echo-Doppler, 4D Flow MRI provides the 3D Flow velocity field within a volumetric region of interest over the cardiac cycle. This work reviews the most recent advances in the development and application of dedicated image analysis techniques for the assessment of intra-cardiac flow features from 4D Flow MRI.

**Recent Findings:**

Novel image analysis techniques have been developed for extraction of relevant intra-cardiac flow features from 4D Flow MRI, which have been successfully applied in various patient cohorts and volunteer studies. Disturbed flow patterns have been linked with valvular abnormalities and ventricular dysfunction. Recent technical advances have resulted in reduced scan times and improvements in image quality, increasing the potential clinical applicability of 4D Flow MRI.

**Summary:**

4D Flow MRI provides unique capabilities for 3D visualization and quantification of intra-cardiac blood flow. Contemporary knowledge on 4D Flow MRI shows promise for further exploration of the potential use of the technique in research and clinical applications.

## Introduction

Two-dimensional (2D) phase-contrast MRI (PC-MRI) is an established acquisition method in clinical MR protocols for vascular flow quantification by acquiring time-resolved cross-sectional images with velocity encoding in the through-plane direction. Based on the area of the vessel cross-section and the average velocity within the defined region, the instantaneous flow rate and total forward and backward flow during a cardiac cycle can be derived. However, the moving geometry of the heart and the complexity of intra-cardiac flow patterns during systole and diastole make it very challenging to quantify flow using 2D phase-contrast PC-MRI. Time-resolved 3D imaging with velocity information in each of the three spatial dimensions has demonstrated reliability and accuracy in quantification of intra-cardiac flow. 4D Flow MRI provides 3D images with encoding of the velocity magnitude and direction of each voxel within the defined volume throughout the cardiac cycle by acquiring data over multiple cardiac cycles. The 3D velocity information obtained, therefore, describes an average cardiac cycle, and information related to beat-to-beat variations is not provided. More than 15 years ago, researchers have started investigating the feasibility of 4D Flow MRI for intra-cardiac flow analysis [1–3]. Until now, relatively few groups have been active in this area of research, potentially because of limitations in the availability of 4D Flow MRI and the long acquisition times limiting the applicability. However, advances in MR hardware and sequence design have resulted in a significant increase in the use of 4D Flow MRI for vascular and intra-cardiac applications [4]. More recently, consensus statement guidelines on the use of 4D Flow MRI were formulated, which aim to assist understanding of acquisition and analysis methods, and their potential clinical applications with a focus on the heart and greater vessels [5•]. The focus of the current review is to summarize methods used and the applications for visual and quantitative analysis of intra-cardiac flow from 4D Flow MRI.

## Imaging Protocol Considerations

The imaging protocol for whole-heart 4D Flow MRI should be tailored to the specific analysis for which it is being used. Parameters to be considered are the volumetric coverage of the acquisition, velocity encoding sensitivity (VENC) selection, temporal and spatial resolution, and the type of respiratory motion compensation. For the assessment of the complete cardiac cycle, including the phases of early and late diastolic filling, retrospective cardiac gating should be employed, such that velocity images are obtained equally spaced over the complete cardiac cycle. Evaluation of the large-scale flow patterns in the heart requires a spatial resolution equal to or <3 × 3 × 3 mm [5•]. Flow analysis using particle tracing requires sufficiently high temporal resolution. As is typical in MR sequence optimization, a proper trade-off needs to be made in order to find the right balance between the parameters to be optimized. Validation studies should be performed, ideally including phantom experiments, in order to gain insight into the accuracy and precision of the parameters to be derived from the employed 4D Flow sequence.

As the magnitude images which are obtained with the 4D Flow scan are typically of poor quality, additional cine MR imaging is often acquired in multiple views to provide an anatomical reference. This allows visualization and analysis of the velocity data from the 4D Flow acquisition in relation to the cardiac motion and anatomy. The number of frames reconstructed from the cine scans should ideally be equal to that of the 4D Flow scan. A disadvantage of such multi-sequence approach is that as a result of patient motion and heart rate variation between scans additional post-processing may be required to correct for image misalignment between sequences. Hsiao et al. have proposed the use of an accelerated post-contrast 4D Flow sequence generating velocity information in the three spatial dimensions along with diagnostic quality anatomical images [6]. They could demonstrate that using this approach reliable assessment of ventricular dimensions in addition to valvular flow quantification can be performed in a single acquisition [7].

## Pre-processing and Data Verification

The obtained image data may require data pre-processing before reliable analyses can be performed. Correction methods have been described for potential errors in the velocity data including velocity aliasing and phase offset errors due to Eddy currents, Maxwell terms, and gradient non-linearity [8–10]. Depending on the MR system used, the scanner software may apply these correction methods in the reconstruction software. Careful evaluation of the data is required as, depending on the analysis performed, small errors in the data may lead to large discrepancies. Visual inspection of the raw velocity images of the three velocity components may reveal velocity aliasing artifacts. Automated phase unwrapping algorithms have been developed to correct phase wrapping artifacts [11]. In order to use the additionally acquired cine MR acquisitions which can be used as an anatomical reference for the velocity data from the 4D Flow scan, misalignment between the cine MR data and the 4D Flow data should be corrected for. Once corrected for misalignment, the cine MRI data facilitate defining anatomical regions and velocity information within defined regions of interest can be interrogated in conjunction with cardiac anatomy. In case cine MRI and 4D Flow acquisition are both obtained using the same breathing motion compensation technique, correction for image misalignment may not be needed. The absence of visually apparent data quality issues does not guarantee that the velocity data are reliable. Based on the conservation of mass principle, additional quantitative verification steps are recommended to further assess the reliability of the data. One such test is the verification of the consistency in net aortic and pulmonary artery outflow which should be equal in the absence of shunts. Another useful check is the comparison of aortic stroke volume as derived from the 4D Flow acquisition with flow assessment from a validated 2D phase-contrast scan. Comparing data from 4D Flow MRI with conventional 2D phase-contrast has also been proposed to evaluate the accuracy of peak velocity measurements [12].

## Data Visualization

Visual analysis is typically the first step in the evaluation of an intra-cardiac 4D Flow acquisition. However, the complexity and enormous size of a typical whole-heart 4D Flow dataset poses challenges for effective visual data interpretation. Conventional 3D workstations providing visualization techniques such as volume rendering are of limited value. Nevertheless, for various reasons, visualization of the data is of utmost importance as it can help provide quick clinically relevant insight into the presence of a particular pathology. Additionally, visualization is needed in the process of subsequent quantitative analysis.

### Color Coding

**Fig. 1:**
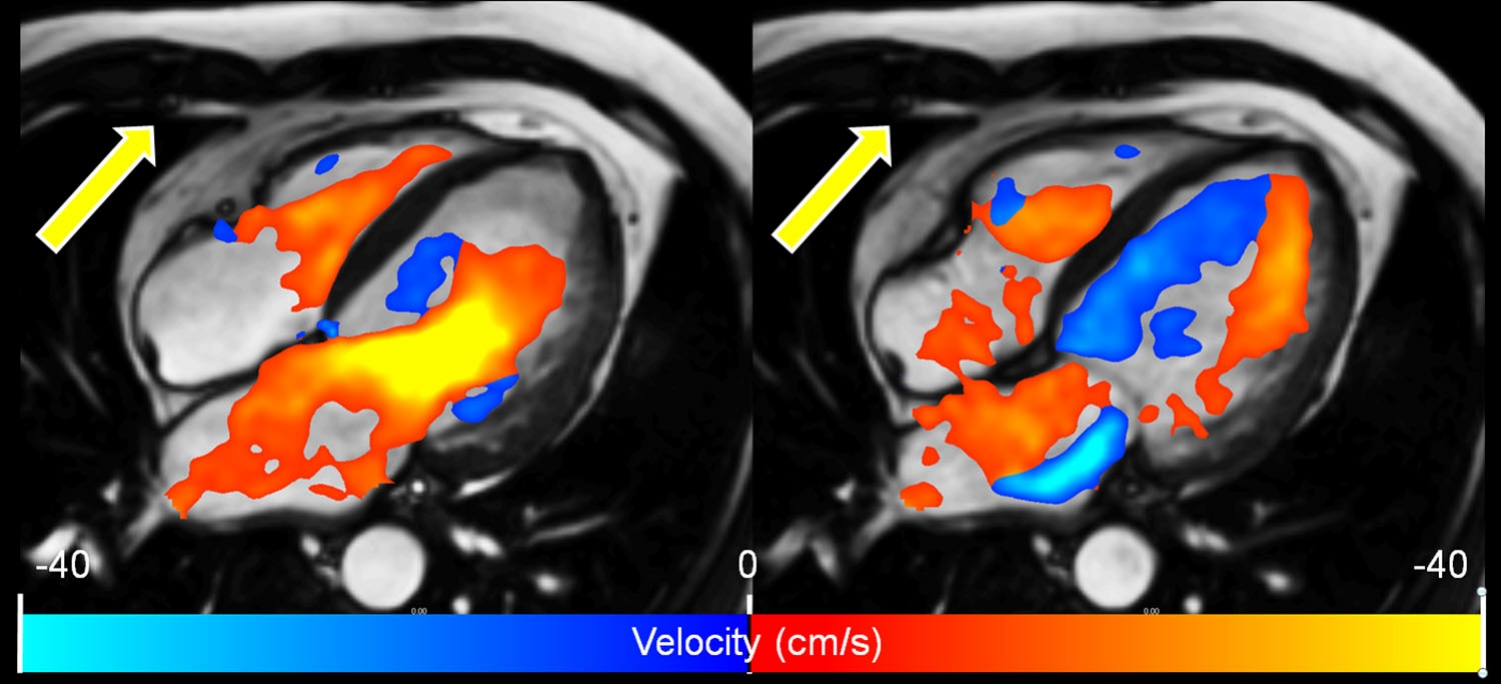
Long-axis view obtained by conventional balanced FFE with color overlay derived from 4D Flow MRI. The coloring applied encodes for the magnitude of velocity in the direction of the indicated *arrow*, simulating Color Doppler flow imaging. The *left panel* shows the moment of peak early LV filling. The *right panel*, showing a systolic phase, clearly reveals the regurgitant jet distal to the mitral valve in the left atrium

The scanned 3D volume can be reformatted into user-defined 2D views which can be color coded according to a certain flow velocity parameter, such as the instantaneous through-plane velocity. Color coding can also be applied as an overlay on other 2D views obtained within the same MR examination. This has the advantage that high-quality anatomical information from standard cine MRI can be combined with flow information from the 4D Flow acquisition. In general, color coding can be of use for the visualization of any instantaneous scalar value that can be derived at a voxel level from the 3D velocity information. Examples of such parameters are the velocity component along a selected direction, velocity magnitude, (turbulent) kinetic energy, and vorticity. Figure 1 shows an example of the use of a color overlay to visualize trans-mitral flow in a way similar as in Color Doppler ultrasound.

### 2D-Velocity Vector Display

**Fig. 2:**
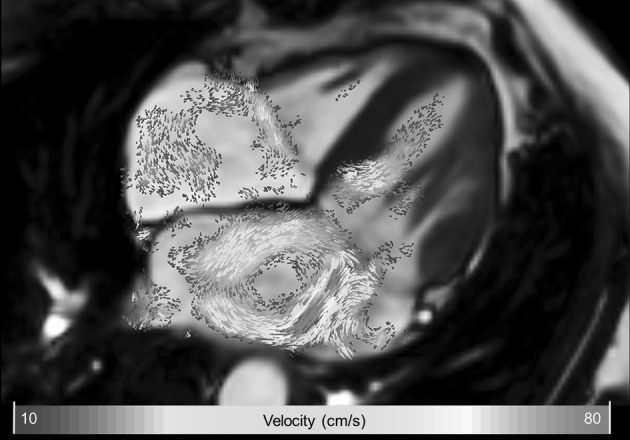
Example of 2D velocity vector display in a patient with mitral valve regurgitation. Systolic four-chamber image showing a central high-velocity jet in the left atrium resulting in a clockwise recirculating flow in the left atrium

The magnitude and direction of velocity can be visualized in 2D or 3D using vector display, i.e., using small arrows or line segments indicating the local blood velocity direction and magnitude. The generated vectors are presented parallel to the direction of velocity and the length and/or coloring of the vector can be used to indicate the velocity magnitude. Displaying a vector for every pixel in 2D or every voxel in 3D will result in an overlap of vectors, which will render the vector visualization of little use. The solution to this is to enforce a certain distance between vectors by using either a fixed distance, or only display vectors in particular regions within the image. A velocity vector display can be useful for qualitative assessment of LV inflow and outflow direction, stenotic jet direction, and assessment of regions of recirculating flow. Figure 2 demonstrates an example of using 2D velocity vector display in a patient with mitral valve regurgitation.

### 2D Streamline Display

**Fig. 3:**
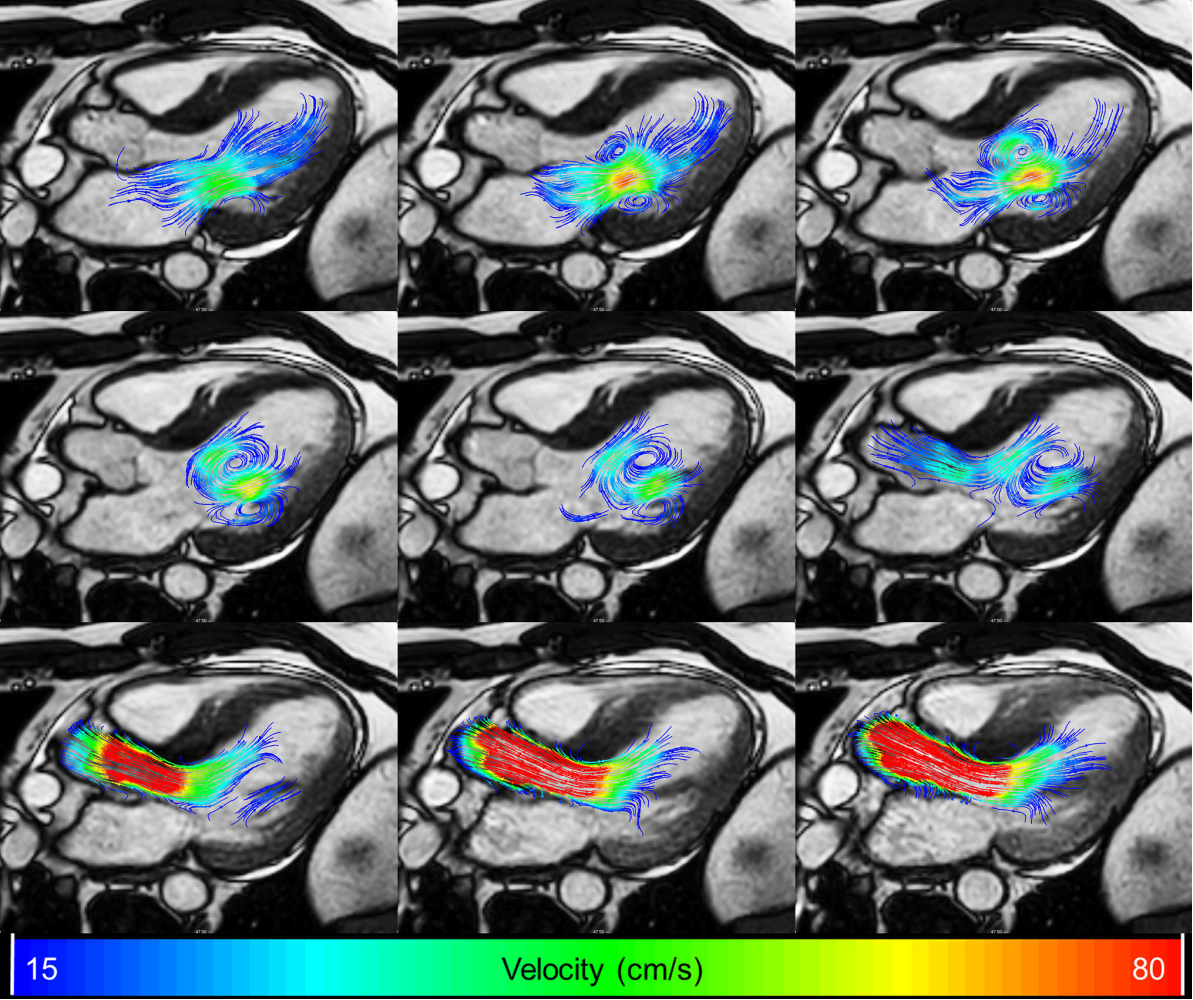
Left ventricular outflow tract view with 2D streamlines as overlay. *Left* moment of late diastolic filling. The presence of a 3D vortex ring can be seen as two counter-rotating recirculating flow regions distal to the mitral valve leaflets. *Middle* end-diastolic moment showing preserved rotational flow. *Right* early systolic moment showing redirection of flow toward the left ventricular outflow tract

Streamlines are curved lines which are locally tangent to the velocity direction. It provides information about the instantaneous velocity field. The displayed line curves should not mistakenly be misinterpreted as flow path lines. Like the vector display method, streamline display is a useful technique to indicate the inflow and outflow directions and is particularly helpful in identifying regions of flow recirculation. Visual cluttering can be minimized by limiting the maximum length of the streamlines, or by generating the streamlines from seed points in the data at a certain interval, or locations exceeding a certain velocity. Figure 3 shows an example of 2D streamline display indicating the flow evolution in the LV from diastole to systole.

### Three-Dimensional Visualization

**Fig. 4:**
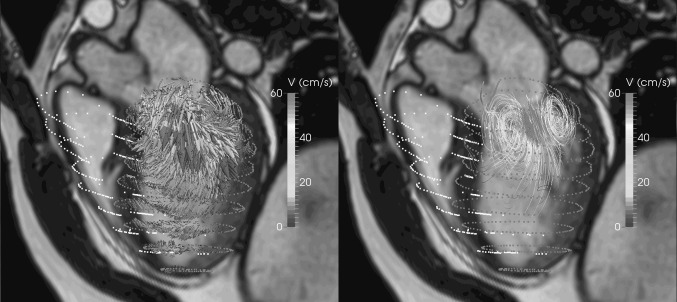
Three-dimensional visualization of intra-cardiac flow used to show mitral inflow velocity at the moment of peak filling. *Left* vector display. *Right* streamline display with seeds defined in spherical region with a radius of 15 mm centered at the mitral valve annulus. LV endocardial surface is displayed with * red dots* and RV endocardial surface with * yellow dots*. The LV outflow tract cine view image is shown in the background as anatomical reference (Color figure online)

The use of 3D visualization techniques is especially useful if used in an interactive and or a dynamic manner. Potentially, all details present in the 3D velocity data can be inspected, but due to data cluttering interactive modification of viewing angle, visualization settings, or volume cropping may be required. Figure 4 shows an example of mitral inflow visualized as 3D vectors and as 3D streamline display. It is obvious that compared to the vector display, the streamline display is better capable of revealing the vortical flow pattern at the mitral valve tips. Due to the overlap of the velocity vectors in the vector display, the vortical flow pattern is less well appreciated with vector display.

## Quantification Techniques

### Flow Quantification Using Retrospectively Defined Measurement Planes

**Fig. 5:**
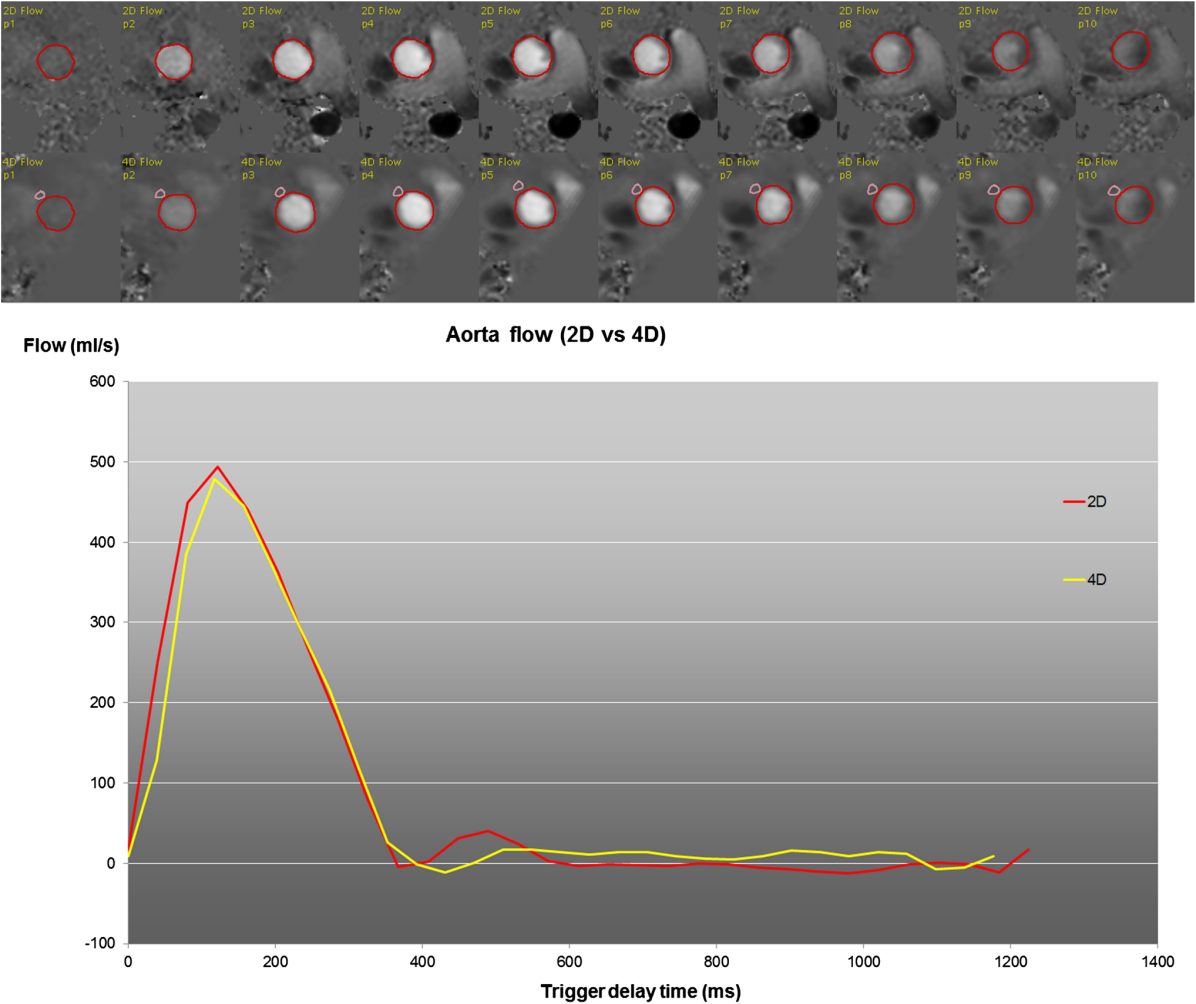
Example of aortic flow curves of a healthy subject derived from conventional 2D phase-contrast MRI (2D Flow) and from a reformatted 4D Flow acquisition. The 4D Flow acquisition was reformatted into a through-plane encoded view, identical to the corresponding 2D Flow acquisition. The top two rows depict the systolic through-plane velocity-encoded images (the first 10 frames out of 30) for 2D Flow and 4D Flow MRI, respectively. Contours are defined around the aortic lumen (shown in *red*). In the 4D Flow-derived images, an additional contour is defined in a region of tissue adjacent to the aorta for velocity offset correction. A good agreement is observed between the two flow curves. The stroke volume derived from 2D Flow is 105 ml and from 4D Flow is 103 ml (Color figure online)

The scanned 3D volume can be retrospectively reformatted into 2D image planes with through-plane velocity encoding to allow flow quantification similar to conventional 2D phase-contrast imaging. Retrospective definition of such measurement planes has several advantages compared to the conventional approach of acquiring individual 2D phase-contrast scans with through-plane velocity encoding. The position and orientation of the reformat planes can be adjusted to the flow direction and multiple measurement planes can be defined from a single acquisition. With all measurements derived from the same scan, heart rate variations between scans will not degrade measurement consistency, which is relevant for the assessment of left-to-right shunts. Figure 5 shows an example of an aortic flow measurement derived from 4D Flow MRI compared to conventional 2D phase-contrast MRI. If needed, the plane definition can be adjusted per individual image frame which is needed for accurate trans-mitral and trans-tricuspid valvular flow quantification as has been demonstrated in studies by Westenberg and Roes et al. [1, 13]. In these studies, a retrospective valve tracking approach was used to accurately position the measurement plane according to the position and orientation of the valve annulus throughout the cardiac cycle. The consistency of net flow through the inflow and outflow valves was significantly improved using the retrospective valve tracking method with 4D Flow MRI. Optimizing the image plane position and orientation per individual phase is also essential for accurate quantification of regurgitant flow as the direction of the regurgitant jet may vary considerably [1]. In a recent study by Calkoen et al., the investigators explored the optimal positioning of the 2D measurement planes for quantification of trans-mitral flow in patients with corrected atrioventricular septal defect [14]. They found that for inflow quantification, the measurement plane should be positioned at the location of maximum inflow velocity perpendicular to the inflow direction, as can be assessed from streamline visualization.

### Particle Tracing Quantification

Particle tracing aims at assessing the 3D trajectory of a blood volume through the heart chambers over the cardiac cycle [15]. It requires the selection of a region within the blood pool from which synthetic particles are released at a particular time point within the cardiac cycle. Typically, the dimension of the chosen particles equals the voxel size of the 4D Flow acquisition, but any other particle definition can be used. The path of traveling is derived by integration of the velocity over time of each individual particle. The generated paths can be visualized dynamically as particle movies, or as a static image by showing the complete length of the particle trace. When all voxels within the LV cavity at end diastole are used as particles and particle tracing is performed until the end-systolic moment, several qualitative and quantitative assessments can be made. Firstly, it tells which part of the blood volume in the end-diastolic phase is ejected into the aorta during systole. Secondly, the percentage of particles that enter the aorta could potentially be used as an alternative method to derive the ejection fraction. An alternative method of particle tracing is to perform tracing backward in time. Using the end-diastolic blood volume in the ventricular cavity as particles and performing backward tracing, the computed particle traces provide insight into the trajectory of early and late diastolic inflow volume. The combination of forward and backward particle tracing has been used by a number of researchers to enable blood flow component classification [16]. Table 1 summarizes the classification rules applied to define blood flow components into either *direct flow*, *retained inflow*, *delayed ejection*, or *residual volume*. Several authors have applied this approach to study blood transportation efficiency in healthy individuals and various patient populations [17, 18]. Efficient blood transportation is assumed to be associated with a higher percentage of direct flow. Particle tracing with flow component analysis has also been successfully applied to the right heart [19, 20]. In a study by Frederiksson et al., flow component analysis was performed for both the LV and RV [19]. It was shown that the percentage of direct flow is higher and the percentage of residual volume is lower for the RV compared to the LV, while residual volume and delayed ejection volume were similar. In a later study, it was demonstrated that, based on RV flow component analysis, in patients with ischemic LV disease, functional impairment in RV function is present [20].

**Table 1:** Flow component classification. Flow component classification rules for labeling particles used in particle tracing flow analysis (derived from Eriksson et al. [18])

Flow component	Definition
Direct flow	Blood that enters the LV during diastole and leaves the LV during systole in the analyzed heart beat; component of both inflow and ejected volume
Retained inflow	Blood that enters the LV during diastole but does not leave during systole in the analyzed heart beat; component of inflow volume only
Delayed ejection flow	Blood that starts and resides inside the LV during diastole and leaves during systole in the analyzed heart beat; component of ejected volume only
Residual volume	Blood that resides within the LV for at least two cardiac cycles; not a component of inflow or ejected volume

**Fig. 6:**
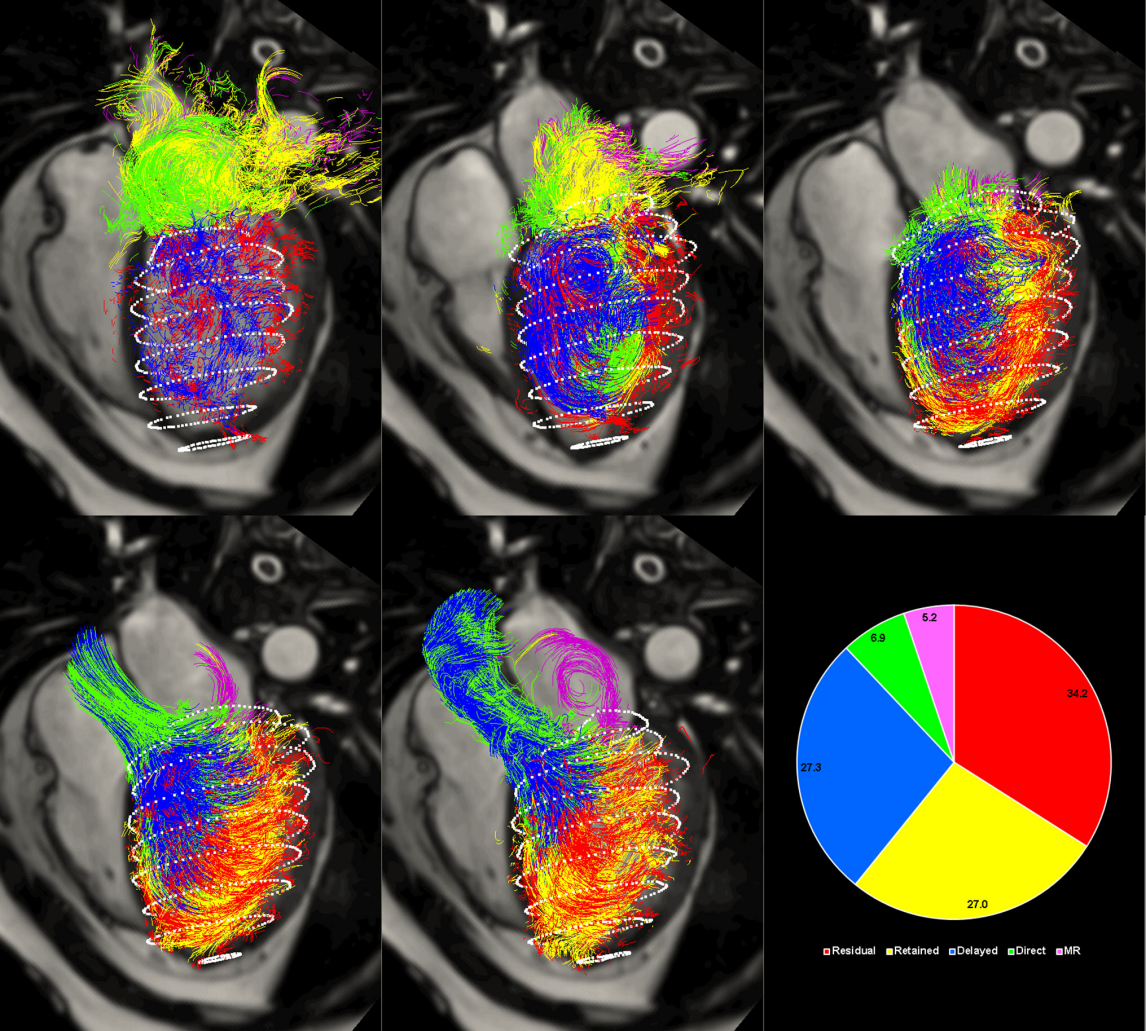
Result of flow component analysis using particle tracing of a patient with mitral valve regurgitation. Displayed path lines are color coded according to flow component classification. *Top row from left to right* start of early filling, peak early filling, end diastole; *Bottom row* early systole, late systole, pie depicting percentage of LV for each of the five defined flow components. Regurgitant jet results in recirculating flow pattern in the left atrium

In the implementation of the flow component characterization according to the definition as summarized in Table 1, the most basal LV or RV plane is often used to evaluate whether a particle has left the ventricular cavity during systole. However, in case of mitral or tricuspid regurgitation, particles that appear above the basal plane during systole may be either related to forward or backward flow. With a more meticulous way of differentiating forward from regurgitant flow, regurgitant flow can also be detected using particle tracing analysis. Figure 6 shows an example of flow component analysis in a patient with moderate mitral valve regurgitation. It is shown that the regurgitant jet results in a recirculating flow pattern in the left atrium.

### Kinetic Energy Quantification

**Fig. 7:**
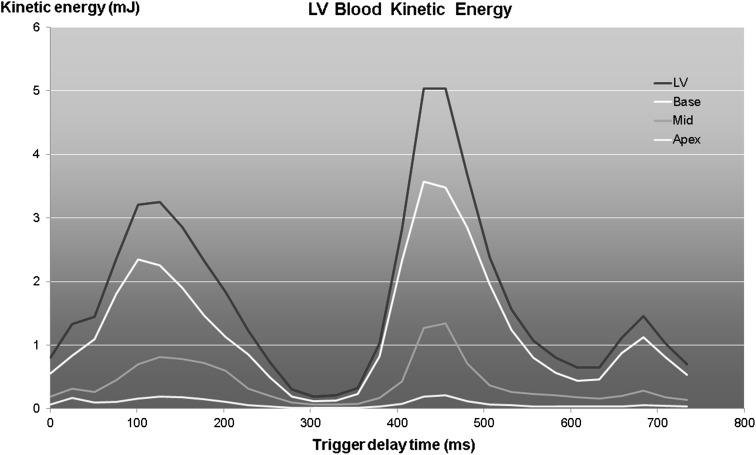
Temporal evolution of kinetic energy of blood in the LV for a healthy subject computed for multiple levels of the LV. The * red curve* shows the total LV kinetic energy

The kinetic energy (KE) of a moving particle with mass $m$ and velocity $v$ can be computed using the formula $E = \frac{1}{2}m \times v^{2}$. With this formula, the KE of the flowing blood in the heart chambers can be computed by summing the KE of each individual voxel within the cavity. Carlsson et al. studied the KE in the left and right ventricle (RV) in healthy individuals over the complete cardiac cycle [21]. Three distinct peaks in KE were observed for both the LV and RV during systole, early and late diastole. The systolic peak was higher for the RV, while the early diastolic peak was higher for the LV. The late diastolic peaks were significantly lower than the other two peaks. In a later study, Kanski et al. performed LV KE quantification in heart failure patients [22]. The KE curves in patients were found to be markedly different from controls suggesting a potential value of KE assessment in evaluating heart failure patients. However, only poor agreement was found between KE time curve patterns and degree of diastolic dysfunction, and no association was observed with NYHA classification. Al-Wakeel and coworkers demonstrated alteration in the pattern of LV kinetic energy in patients before and after mitral valve surgery [23]. Wong et al. studied age-related changes in ventricular diastolic energetics [24•]. They found a progressive decrease in peak diastolic KE with age. An example of the pattern of KE in the LV at multiple ventricular levels is shown in Fig. 7. As blood particles travel from the atrium through the ventricle into outflow, the velocity, and thus the kinetic energy of a particle, will vary. Several studies have reported on KE evaluation of blood particles according to flow component classification [16, 25]. In a study by Bolger et al., it was shown that 19 % of the kinetic energy of inflowing LV particles was preserved until the end of diastole, while this was only 5 % in a DCM patient [16].

### Analysis of Vortical Flow

For optimized cardiac pump function, it would be advantageous if the KE of inflowing blood flow is preserved until the end of diastole and would contribute to energy-efficient ejection of blood during systole. The development of vortical flow patterns in the heart during diastole is believed to play an important role in the process of flow redirection and contributes to cardiac pump efficiency [26, 27•]. Töger et al. proposed the use of Lagrangian Coherent Structures to derive the vortical flow volume within LV cavity during diastole [28]. In their experiments, they found the volume of vortical flow in healthy individuals to encompass 52 % of the total LV volume. Elbaz et al. used the Lambda-2 method for the detection of the ring-like vortex core structures that develop distal to the mitral valve annulus during early and late diastolic filling [29]. In healthy subjects, it was found that the vortex cores were more circular in shape during early filling compared to late filling and the shape of the vortex ring core was found to correlate with the shape of the mitral valve annulus. In a cohort of 32 patients with a corrected atrioventricular septal defect, which have abnormal mitral valve geometry, Calkoen et al. applied the same Lambda-2-based vortex core detection method and found a strong association between vortex core presence and shape and mitral valve shape and LV inflow direction [30].

## Discussion

Over the past 15 years, 4D Flow MRI has developed into a technique suitable for research use, but also with high potential for clinical application. The interaction between myocardial dynamics and intra-cardiac blood flow can now be studied with 4D Flow MRI in an individual subject. Moreover, this allows verification and refinements of concepts from the field of computational flow engineering to enhance our insight into normal and abnormal cardiac physiology. 4D Flow MRI has already been shown to be applicable for clinical application in the assessment of transvalvular flow and for a comprehensive evaluation of patients with heart disease. Further improvements in 4D Flow acquisition and data analysis are highly desirable to make the imaging and analysis more time efficient and easier to use. Additional research is also required to evaluate the added value of 4D Flow MRI in clinical patient management.

## References

[1] Westenberg JJ, Roes SD, Ajmone Marsan N, Binnendijk NM, Doornbos J, Bax JJ, Reiber JH, de Roos A, van der Geest RJ (2008). Mitral valve and tricuspid valve blood flow: accurate quantification with 3D velocity-encoded MR imaging with retrospective valve tracking. Radiology.

[2] Wigström L, Sjöqvist L, Wranne B (1996). Temporally resolved 3D phase-contrast imaging. Magn Reson Med.

[3] Eriksson J, Carlhall CJ, Dyverfeldt P, Engvall J, Bolger AF, Ebbers T (2010). Semiautomatic quantification of 4D left ventricular blood flow. J Cardiovasc Magn Reson.

[4] Markl M, Kilner PJ, Ebbers T (2011). Comprehensive 4D velocity mapping of the heart and great vessels by cardiovascular magnetic resonance. J Cardiovasc Magn Reson.

[5] • Dyverfeldt P, Bissell M, Barker AJ, Bolger AF, Carlhäll CJ, Ebbers T, Francios CJ, Frydrychowicz A, Geiger J, Giese D, Hope MD, Kilner PJ, Kozerke S, Myerson S, Neubauer S, Wieben O, Markl M. 4D flow cardiovascular magnetic resonance consensus statement. J Cardiovasc Magn Reson 2015;17:22. *Consensus statement paper providing guidelines for 4D Flow MRI acquisition and analysis methods for evaluation of the heart and greater vessels.* 10.1186/s12968-015-0174-5 PMC4530492 26257141

[6] Hsiao A, Tariq U, Alley MT, Lustig M, Vasanawala SS (2015). Inlet and outlet valve flow and regurgitant volume may be directly and reliably quantified with accelerated, volumetric phase-contrast MRI. J Magn Reson Imaging.

[7] Hanneman K, Kino A, Cheng JY, Alley MT, Vasanawala SS (2016). Assessment of the precision and reproducibility of ventricular volume, function, and mass measurements with ferumoxytol-enhanced 4D flow MRI. J Magn Reson Imaging..

[8] Walker PG, Cranney GB, Scheidegger MB, Waseleski G, Pohost GM, Yoganathan AP (1993). Semiautomated method for noise reduction and background phase error correction in MR phase velocity data. J Magn Reson Imaging.

[9] Bernstein MA, Zhou XJ, Polzin JA, King KF, Ganin A, Pelc NJ, Glover GH (1998). Concomitant gradient terms in phase contrast MR: analysis and correction. Magn Reson Med.

[10] Markl M, Bammer R, Alley MT (2003). Generalized reconstruction of phase contrast MRI: analysis and correction of the effect of gradient field distortions. Magn Reson Med.

[11] Xiang QS (1995). Temporal phase unwrapping for CINE velocity imaging. J Magn Reson Imaging.

[12] Carlsson M, Töger J, Kanski M, Bloch KM, Ståhlberg F, Heiberg E, Arheden H (2011). Quantification and visualization of cardiovascular 4D velocity mapping accelerated with parallel imaging or k-t BLAST: head to head comparison and validation at 1.5 T and 3 T. J Cardiovasc Magn Reson.

[13] Roes SD, Hammer S, van der Geest RJ, Marsan NA, Bax JJ, Lamb HJ, Reiber JH, de Roos A, Westenberg JJ (2009). Flow assessment through four heart valves simultaneously using 3-dimensional 3-directional velocity-encoded magnetic resonance imaging with retrospective valve tracking in healthy volunteers and patients with valvular regurgitation. Invest Radiol.

[14] Calkoen EE, Roest AA, Kroft LJ, van der Geest RJ, Jongbloed MR, van den Boogaard PJ, Blom NA, Hazekamp MG, de Roos A, Westenberg JJ (2015). Characterization and improved quantification of left ventricular inflow using streamline visualization with 4D Flow MRI in healthy controls and patients after atrioventricular septal defect correction. J Magn Reson Imaging.

[15] Wigström L, Ebbers T, Fyrenius A, Karlsson M, Engvall J, Wranne B, Bolger AF (1999). Particle trace visualization of intracardiac flow using time-resolved 3D phase contrast MRI. Magn Reson Med.

[16] Bolger AF, Heiberg E, Karlsson M, Wigström L, Engvall J, Sigfridsson A, Ebbers T, Kvitting JP, Carlhäll CJ, Wranne B (2007). Transit of blood flow through the human left ventricle mapped by cardiovascular magnetic resonance. J Cardiovasc Magn Reson.

[17] Eriksson J, Carlhäll CJ, Dyverfeldt P, Engvall J, Bolger AF, Ebbers T (2010). Semi-automatic quantification of 4D left ventricular blood flow. J Cardiovasc Magn Reson.

[18] Eriksson J, Bolger AF, Ebbers T, Carlhäll CJ (2013). Four-dimensional blood flow-specific markers of LV dysfunction in dilated cardiomyopathy. Eur Heart J Cardiovasc Imaging..

[19] Fredriksson AG, Zajac J, Eriksson J, Dyverfeldt P, Bolger AF, Ebbers T, Carlhäll CJ (2011). 4-D blood flow in the human right ventricle. Am J Physiol.

[20] Fredriksson AG, Svalbring E, Eriksson J, Dyverfeldt P, Alehagen U, Engvall J, Ebbers T, Carlhäll CJ (2016). 4D flow MRI can detect subtle right ventricular dysfunction in primary left ventricular disease. J Magn Reson Imaging.

[21] Carlsson M, Heiberg E, Töger J, Arheden H (2012). Quantification of left and right ventricular kinetic energy using four-dimensional intracardiac magnetic resonance imaging flow measurements. Am J Physiol.

[22] Kanski M, Arvidsson PM, Töger J, Borgquist R, Heiberg E, Carlsson M, Arheden H (2015). Left ventricular fluid kinetic energy time curves in heart failure from cardiovascular magnetic resonance 4D flow data. J Cardiovasc Magn Reson.

[23] Al-Wakeel N, Fernandes JF, Amiri A, Siniawski H, Goubergrits L, Berger F, Kuehne T (2015). Hemodynamic and energetic aspects of the left ventricle in patients with mitral regurgitation before and after mitral valve surgery. J Magn Reson Imaging.

[24] • Wong J, Chabiniok R, de Vecchi A, Dedieu N, Sammut E, Schaeffter T, Razavi R. Age-related changes in intra-ventricular kinetic energy: a physiological or pathological adaptation? Am J Physiol Heart Circ Physiol. 2016; 310(6):H747–755. *Kinetic energy calculation was performed in the left ventricle from 4D Flow MRI in healthy subjects of different age ranges and patients with left ventricular dysfunction. Age related changes in kinetic energy were observed in healthy subjects. Peak diastolic kinetic energy in the oldest subject was shown to be comparable to those in patient with LV dysfunction.*

[25] Eriksson J, Bolger AF, Ebbers T, Carlhäll CJ (2013). Four-dimensional blood flow-specific markers of LV dysfunction in dilated cardiomyopathy. Eur Heart J.

[26] Kilner PJ, Yang GZ, Wilkes AJ, Mohiaddin RH, Firmin DN, Yacoub MH (2000). Asymmetric redirection of flow through the heart. Nature.

[27] • Pedrizzetti G, La Canna G, Alfieri O, Tonti G. The vortex—an early predictor of cardiovascular outcome? Nat. Rev. Cardiol 2014;11(9):545–553. *The role cardiac fluid dynamics and in particular vortex formation in the heart is described and proposed as a new potential marker that can be used for cardiac risk stratification.* 10.1038/nrcardio.2014.75 24889521

[28] Töger J, Kanski M, Carlsson M, Kovács SJ, Söderlind G, Arheden H, Heiberg E (2012). Vortex ring formation in the left ventricle of the heart: analysis by 4D flow MRI and Lagrangian coherent structures. Ann Biomed Eng.

[29] Elbaz MS, Calkoen EE, Westenberg JJ, Lelieveldt BP, Roest AA, van der Geest RJ (2014). Vortex flow during early and late left ventricular filling in normal subjects: quantitative characterization using retrospectively-gated 4D flow cardiovascular magnetic resonance and three-dimensional vortex core analysis. J Cardiovasc Magn Reson.

[30] Calkoen EE, Elbaz MS, Westenberg JJ, Kroft LJ, Hazekamp MG, Roest AA, van der Geest RJ (2015). Altered left ventricular vortex ring formation by 4-dimensional flow magnetic resonance imaging after repair of atrioventricular septal defects. J Thorac Cardiovasc Surg..

